# “Omics” insights into plastid behavior toward improved carotenoid accumulation

**DOI:** 10.3389/fpls.2022.1001756

**Published:** 2022-10-06

**Authors:** Yuanyuan Li, Yue Jian, Yuanyu Mao, Fanliang Meng, Zhiyong Shao, Tonglin Wang, Jirong Zheng, Qiaomei Wang, Lihong Liu

**Affiliations:** ^1^ Key Laboratory of Horticultural Plant Growth and Development, Ministry of Agriculture, Department of Horticulture, Zhejiang University, Hangzhou, China; ^2^ Hangzhou Academy of Agricultural Sciences, Hangzhou, China

**Keywords:** carotenoid, plastids, omics, chromoplast, retrograde signaling

## Abstract

Plastids are a group of diverse organelles with conserved carotenoids synthesizing and sequestering functions in plants. They optimize the carotenoid composition and content in response to developmental transitions and environmental stimuli. In this review, we describe the turbulence and reforming of transcripts, proteins, and metabolic pathways for carotenoid metabolism and storage in various plastid types upon organogenesis and external influences, which have been studied using approaches including genomics, transcriptomics, proteomics, and metabonomics. Meanwhile, the coordination of plastid signaling and carotenoid metabolism including the effects of disturbed carotenoid biosynthesis on plastid morphology and function are also discussed. The “omics” insight extends our understanding of the interaction between plastids and carotenoids and provides significant implications for designing strategies for carotenoid-biofortified crops.

## Introduction

Carotenoids are a group of isoprenoid compounds distributed widely in all photosynthetic organisms (including plants, algae, bacteria) and some non-photosynthetic bacteria and fungi (Sun and Li, 2020; Sun et al., 2022). In plants, carotenoids have both primary (essential) and secondary (specialized) roles (Li et al., 2016b). In green tissues, photosynthesis and photoprotection require carotenoids such as lutein, β-carotene, and violaxanthin (Jia et al., 2022). A majority of carotenoids and their volatile cleavage products give fruits and flowers a distinctive color and flavor to attract pollinating animals (Sandmann, 2021). Meanwhile, carotenoids provide precursors for the biosynthesis of *phytohormones abscisic acids* (ABA) and *strigolactones* (SLs) and serve as communication signals with environment as specialized metabolites (Chen et al., 2020). Besides their central roles in plants, carotenoids also play essential roles in human nutrition and health (Torres-Montilla and Rodriguez-Concepcion, 2021). In addition to providing dietary sources of provitamin A (Li et al., 2016a; Giuliano, 2017), they serve as antioxidants that reduce the risk of chronic diseases such as cardiovascular disease, cancers, and age-related eye diseases (Rodriguez-Concepcion et al., 2018; Quian-Ulloa and Stange, 2021).

**Figure 1:**
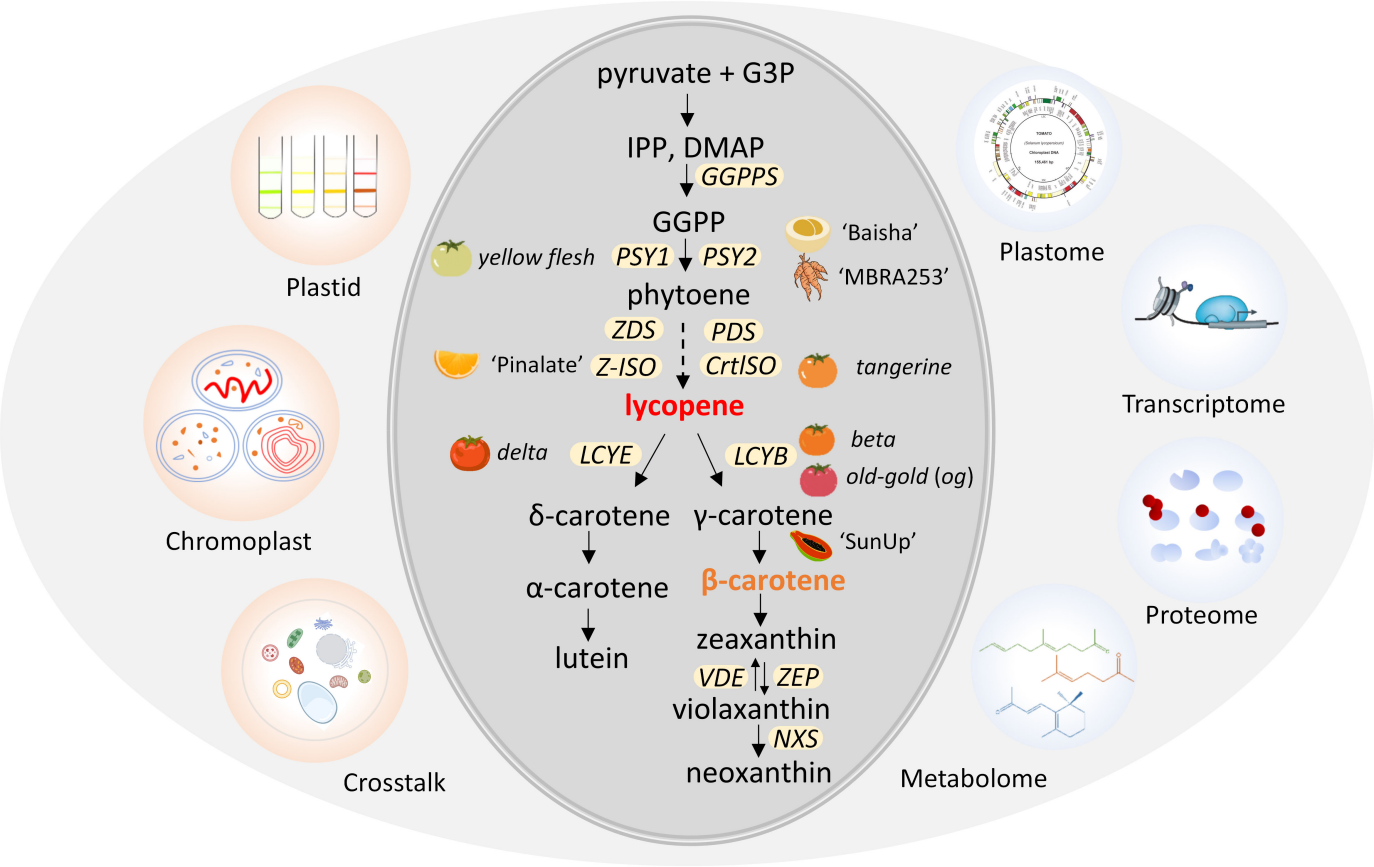
Schematic diagram of carotenoids metabolic pathway and omics in plant plastid. Plastids can be isolated for futher biochemical and molecular characterization *via* omics, including chromoplast with diverse structures. Plastids possess unique crosstalk mechanisms to synthesize and accumulate massive amounts of carotenoids. Mutants or varieties associated with genes for carotenoid biosynthesis are indicated next to the gene. G3P, glyceraldehyde 3-phosphate; IPP, isopentenyl diphosphate; DMAP, dimethylallyl diphosphate; GGPP, geranylgeranyl diphosphate. The yellow ellipses represent carotenoid pathway genes localized in plastids. G*GPPS*, *geranylgeranyl pyrophosphate synthase*; *PSY*, *phytoene synthase*; *PDS*, *phytoene desaturase*; *Z-ISO*, *ζ-carotene isomerase*; *CrtISO*, *carotene isomerase*; *LCYB*, *lycopene β-cyclase*; *LCYE*, *lycopene ϵ-cyclase*; *VDE*, *violaxanthin de-epoxidase*; *ZEP*, *zeaxanthin epoxidase*; *NXS*, *neoxanthin synthase gene*; ‘Baisha’, loquat (*Eriobotrya japonica)*; ‘MBRA253’, cassava (*Manihot esculenta)*; ‘Pinalate’, sweet orange (*Citrus sinensis*); ‘SunUp’, Papaya, (*Carica papaya*).

The carotenoid metabolic pathway can be divided into two parts, lycopene production and cyclization (**Figure 1**). The first bottleneck reaction is bonding two molecules of *geranylgeranyl diphosphate* (GGPP) by *phytoene synthase* (PSY) to synthesize the colorless 15-cis-phytoene (Zhou et al., 2022). Then, 15-cis-phytoene is converted into red-colored all-trans lycopene by sequential desaturation and isomerization reactions catalyzed by enzymes PDS, Z-ISO, ZDS and CrtISO (Wurbs et al., 2007). The hydroxylation and epoxidation of the rings in these carotenes generates yellow xanthophylls of lutein in the α-branch by *lycopene cyclase* (LCYE) and zeaxanthin in the β-branch by LCYB (Garcia-Plazaola et al., 2007). Following hydroxylation of α-carotene and β-carotene by two heme hydroxylases (LUT1/CYP97c1 and LUT5/CYP97a3) and two non-heme carotene hydroxylases (BCH1 and BCH2), yellow xanthophylls of lutein in the α-branch and zeaxanthin in the β-branch are synthesized (Cazzonelli et al., 2020; Sandmann, 2021). Zeaxanthin is epoxidized by *zeaxanthin epoxidase* (ZEP) and de-epoxidized by *violaxanthin de epoxidase* (VDE), forming the xanthophyll cycle to protect plants from photo damage (Galpaz et al., 2008; Jia et al., 2022). In the final step, *neoxanthin synthase* (NXS) converts violaxanthin to neoxanthin (Aluru et al., 2006; Galpaz et al., 2008; Llorente et al., 2017). A variety of species-specific carotenoids are produced by further modifications of carotenes and xanthophylls (Park et al., 2002; Li et al., 2016a). *Carotenoid cleavage dioxygenases* (CCDs) and *9-cis-epoxycarotenoid dioxygenases* (NCEDs) catalyze carotenoids to apocarotenoid, which can serve as cellular signaling molecules (Priya et al., 2019). The genes and enzymes involved in carotenoid biosynthesis have been isolated and well characterized (**Figure 1**).

The plastids in plant cells are responsible for carotenoid biosynthesis and storage (Fish et al., 2022). Apart from proplastids which are undifferentiated plastids, all other types of plastids are capable of producing *carotenoids* (Sadali et al., 2019). The etioplast is considered an intermediate stage in the development of chloroplasts in plants grown in the dark (Quian-Ulloa and Stange, 2021). Etioplasts accumulate relatively small amounts of carotenoids along with the chlorophyll precursor protochlorophyllide within structures called prolamellar bodies (PLBs) (Johnson and Stepien, 2016). All green tissues contain chloroplasts, and abundant carotenoids are reside in chloroplast thylakoid for photosynthesis and photoprotection (Klasek and Inoue, 2016). The chromoplast is a carotenoid-accumulation plastid that contributes to the varied color of the organs (Ling et al., 2021). Comparatively, chromoplasts contain a wide variety of carotenoid compounds that are essential for the nutritional and sensory quality of agricultural products. On the basis of carotenoid-lipoprotein sequestering structures, chromoplasts can be classified as globular, crystalline, membraneous, or tubular to accommodate different compositions of carotenoids (Torres-Montilla and Rodriguez-Concepcion, 2021). Despite the importance of carotenoids in maintaining agricultural value and health, the knowledge on their synthesis and accumulation is still limited. The distinctive functions of diverse plastids shape the regulatory networks of carotenoid metabolism and influence their capacities for carotenoid biosynthesis and sequestration, which results in various amounts and types of carotenoids in plant organs (Choi et al., 2021). A variety of biotechnological strategies have been developed to understand carotenoid metabolism and to enrich plant tissues with carotenoids. The advanced “omics” technologies including genomics, transcriptomics, proteomics, metabolomics, etc. facilitate our understanding of the carotenoid accumulation in the plastids and aid in the discovery of new genes involved in carotenoid metabolism.

## Plastid genomics

### Plastid genome

Plastids are organelles with their own prokaryotic genomes, genetic structure, and gene expression (Fish et al., 2022). For the past two decades, numerous plastid genomes (plastome) have been sequenced, conferring substantial information of their organization and evolution (Tonti-Filippini et al., 2017). Plastome containing approximately 100 genes in a 100-220 kb sequence and relatively small (non-coding) intergenic spacer regions, is the most gene-dense among the three genomes of higher plants (Barkan, 2011; Rogalski et al., 2015). At present, over 7731 chloroplast genome sequences of land plants are uploaded in the NCBI GenBank Organelle Genome Resources (http://www.ncbi.nlm.nih.gov/genome/organelle/), including that of 61 horticultural plants (**Table 1**). Horticultural plant chloroplast genomes are generally conserved in two types of genes, including the genetic system components (i.e. subunits of an RNA polymerase, rRNAs, tRNAs, and ribosomal proteins) and photosynthetic components (i.e. photosystem I, photosystem II, the cytochrome b6/f complex and the ATP synthase) (Liu et al., 2020; He et al., 2022). Genes coding for catalytic subunit of Clp protease (clpP1), Acyl-CoA carboxylase catalytic subunit (accD) and two putative protein import components (Ycf1 and Ycf2) are consistently retained in most plastome (Ma et al., 2017; Bouchnak and van Wijk, 2021; Rodiger et al., 2021). As a result of this conservation, plastid DNA markers are widely used to establish the phylogeny of many plant groups, such as *Rosaceae*, *Solanaceae* and *Cucurbitaceae* (He et al., 2022; Miao et al., 2022; Zhang Z. et al., 2022). Species boundaries, inter-population variation, and gene flow have also been observed using plastid DNA on a much more local scale. Despite the high conservativeness, plastome size varies between species (**Table 1**). A detailed plastome provides cis-elements to be engineered and valuable data for analyzing plastid gene expression in the horticulture plant (Bock, 2022; Chen et al., 2022). The plastid has been selected as a “protein factory” for biotechnology applications on account of its protein production efficiency by plastome (Bharadwaj et al., 2019). However, the regulatory circuits that control the expression of these functions remain to be identified.

**Table 1:** Complete sequence of chloroplast genome from horticultural crops.

Species	Common name	Variety	Accession	Gene	Reference
*Actinidia chinensis*	kiwifruit	Hongyang	NC_026690.1	120	(Chen et al., 2022)
*Ananas comosus*	Pineapple	F153	NC_026220.1	77	(Liu et al., 2016)
*Atropa belladonna*	Belladonna	Ab5	NC_004561.1	85	(Schmitz-Linneweber et al., 2002)
*Brassica juncea*	Zhacai		NC_028272.1	79	(Prabhudas et al., 2016)
*Brassica oleracea*	Broccoli	italica	MH388765.1,	88	(Zhang Z. et al., 2022)
*Calotropis gigantea*	Crown flower		NC_041431.1	82	(Li H. et al., 2016)
*Camellia sinensis*	Tea tree	Qiancha1	OL450428.1	92	(Zhang et al., 2020)
*Capsicum annuum*	Pepper	FS4401	NC_018552.1	86	(Siddique et al., 2006)
*Carnegiea gigantea*	Saguaro cactus		NC_027618.1	67	(Sanderson et al., 2015)
*Catharanthus roseus*	Madagascar periwinkle	NTU2011	NC_021423.1	87	(Ku et al., 2013)
*Citrus sinensis*	Sweet orange	RidgePineapple	NC_008334.1	89	(Bausher et al., 2006)
*Cocos nucifera*	Coconut palm		NC_022417.1	47	(Huang et al., 2013)
*Cucumis melo*	Muskmelon		MT622320.1	87	(Bai et al., 2021)
*Cucumis sativus*	Cucumber	Baekmibaekdadagi	DQ119058	113	(Kim et al., 2006)
*Cucurbita pepo*	pumpkin		NC_038229	84	(Aguirre-Dugua et al., 2019)
*Datura stramonium*	Mandala	BJ018	NC_018117.1	87	(Asaf et al., 2016)
*Daucus carota*	Carrot		NC_008325.1	85	(Ruhlman et al., 2006)
*Dianthus caryophyllus*	Carnation		NC_039650.1	83	(Chen S. et al., 2018)
*Eucommia ulmoides*	Hardy rubber tree		NC_037948.1	89	(Wang W. et al., 2018)
*Fragaria iinumae*	Nogo strawberry		KP718887.1	77	(Govindarajulu et al., 2015)
*Fragaria vesca*	Woodland strawberry		NC_015206.1	85	(Shulaev et al., 2011)
*Glycine max*	Soybean		NC_007942.1	83	(Saski et al., 2005)
*Hordeum vulgare*	Barley	trifurcatum	NC_056985.1	84	(Ren et al., 2021)
*Jasminum nudiflorum*	Winter jasmine		NC_008407.1	85	(Lee et al., 2007)
*Lactuca sativa*	Lettuce	Asparagina Red	NC_007578.1	86	(Liu et al., 2020)
*Lepidium meyenii*	Maca		MT430983.1	85	(Zhou et al., 2017)
*Lilium lancifolium*	Lilies		NC_035589.1	112	(Kim et al., 2017)
*Liriodendron tulipifera*	Magnoliaceae		MK477550.1	84	(Park et al., 2019)
*Malus hupehensis*	Wild apple		NC_040170.1	78	(Zhang et al., 2018)
*Medicago truncatulata*	Barrel medic	Jema Long A-17	NC_003119.8	76	(Saski et al., 2005)
*Morella rubra*	Chinese bayberry		KY476637	83	(Liu et al., 2017)
*Moringa oleifera*	Moringa		NC_041432.1	71	(Lin et al., 2019)
*Musa acuminata*	Banana	Malaccensis	HF677508.1	89	(Martin et al., 2013)
*Nicotiana sylvestris*	Tobacco		NC_007500.1	101	(Yukawa et al., 2006)
*Nicotiana tabacum*	Tobacco	Bright Yellow 4	NC_001879.2	98	(Schmitz-Linneweber et al., 2002)
*Nicotiana tomentosiformis*	Tobacco		NC_007602.1	102	(Asaf et al., 2016)
*Nicotiana undulata*	Tobacco		NC_016068.1	110	(Mehmood et al., 2020)
*Nymphaea alba*	Water lily		NC_006050.1	85	(Goremykin et al., 2004)
*Primula veris*	Cowslip		NC_031428.1	85	(Zhou et al., 2016)
*Prunus avium*	Sweet cherry	Summit	NC_044701.1	77	(Zhao et al., 2019)
*Prunus mume*	Mei	W53	MW755970.1	85	(Huang et al., 2022)
*Prunus persica*	Peach		NC_014697.1	85	(Jansen et al., 2011)
*Prunus yedoensis*	Yoshino cherry	Somei-yoshino	KU985054.1	86	(Cho et al., 2018)
*Pyrus bretschneideri*	Chinese pear	Yali	KX450881.2	81	(Qiao et al., 2016)
*Pyrus communis*	European pear		NC_045336.1	86	(Liu et al., 2019)
*Rosa chinensis*	Chinese rose	spontanea	NC_038102.1	84	(Jian et al., 2018)
*Rosa multiflora*	Many-flowered rose		NC_039989.1	90	(Zhao and Gao, 2020)
*Rosa roxburghii*	Chestnut rose		NC_032038.1	88	(Wang Q. et al., 2018)
*Saccharum* spp.	Sugarcane	Q155	NC_029221.1	82	(Hoang et al., 2015)
*Solanum bulbocastanum*	Solanum nigrum	PT29	NC_007943.1	85	(Chung et al., 2006)
*Solanum lycopersicum*	Tomato	IPA-6	NC_007898.2	114	(Daniell et al., 2006)
*Solanum melongena*	Eggplant		NC_030207.1	85	(Ding et al., 2016)
*Solanum tuberosum*	Potato		NC_008096.2	84	(Chung et al., 2006)
*Spinacia oleracea*	Spinach		NC_002202.1	108	(Schmitz-Linneweber et al., 2001)
*Spinacia oleracea*	Spinach	Monatol	NC_002202.1	96	(Schmitz-Linneweber et al., 2001)
*Vaccinium corymbosum*	Blueberry	Sharpblue	MZ328079.1	100	(Miao et al., 2022)
*Vaccinium macrocarpon*	American cranberry		NC_019616.1	75	(Fajardo et al., 2012)
*Vigna radiata*	Mungbean		NC_013843.1	108	(Tangphatsornruang et al., 2010)
*Vitis vinifera*	Grape		NC_007957.1	61	(Jansen et al., 2006)
*Zingiber officinale*	Ginger		KM213122.1	60	(Vaughn et al., 2014)
*Ziziphus jujuba*	Jujube		NC_030299.1	112	(Ma et al., 2017)

### Plastid gene expression

Plastid genes are highly expressed in chloroplasts but are drastically downregulated in nongreen plastids (Kahlau and Bock, 2008). The genome organization and gene expression in plastids are largely similar to those in bacteria (Zoschke and Bock, 2018). Most plastid genes are operon gene expression with co-transcription pattern. Plastid primary transcripts will be processed successively to generate the mature ones, including cleavage of polycistronic transcripts into oligocistronic or monocistronic isoforms, 5’ and 3’ end processing, intron splicing, and C-to-U editing (Majeran et al., 2012; Bock, 2022). Plastid-encoded RNA polymerase (PEP) and nuclear-encoded plastid RNA polymerase (NEP) transcribe overlapped sets of genes contributing to the complexity of plastid transcription (Koussevitzky et al., 2007). Several plastid genes are predominantly transcribed from PEP or NEP, but many genes have promoters for both RNA polymerases (Pfannschmidt et al., 2015). It is important to understand the regulation and expression of plastid genes through a combination of ChIP-seq and RNA-seq analysis in plastids. The ptChIP-seq can demonstrate the levels of PEP binding to DNA and show the impact of PEP on chloroplast gene expression throu gh a publicly available tool named Plaviso (https://plavisto.mcdb.lsa.umich.edu/) (Palomar et al., 2022).

During tomato (*Solanum lycopersicum*) fruit ripening and concomitant chloroplast-to-chromoplast transformation, minor changes in plastid RNA accumulation were observed. Whereas, most plastid genes expressions are dramatically downregulated in ripening fruits compared with those in leaves and are translationally downregulated during chromoplast development. With the exception of *accD* involved in fatty acid biosynthesis, both transcriptional and translational downregulation are pronounced for photosynthetic genes (Kahlau and Bock, 2008). In kiwifruit (*Actinidia chinensis* cv. Hongyang), several photosynthesis-related genes, as well as most genes associated with the genetic system, were substantially upregulated in green fruits compared to leaves and nearly all plastid genes were significantly downregulated during fruit development (Chen et al., 2022). Although the expression of *psbA* remained unchanged, the level of psbA protein decreased continuously during chloroplast-to-chromoplast transformation (Chen et al., 2022).

### Plastid genome engineering

Plastid genome engineering has evolved in transgene expression (Hasunuma et al., 2008). Current plastid research is focused on plastid transformation for biotechnology applications, namely molecular agriculture that adds new agronomic traits, controls metabolic pathways, enhances insect resistance, and agricultural and horticulture-related species (Hasunuma et al., 2008; Rogalski et al., 2015). Plastid transformation is the process of targeting foreign genes to plastids to obtain plants with new traits. The synthetic operon sequence elements that confer processing into monocistronic mRNAs are a safe strategy, and have been successfully applied to a number of metabolic pathways (Lu et al., 2017). Homologous recombination is required for transformation, which facilitates site-specific alteration of endogenous plastid genes and precise targeted insertion of foreign genes into plastid DNA. The carotenoid pathway engineering of plastid genomes by transgene expression has profound advantages over nuclear transformation, including higher levels of heterologous gene expression, materal inheritance for transgene containment, lack of position effects and gene silencing, and the straightforward multigene engineering (Lelivelt et al., 2005; Hasunuma et al., 2008; Yarra, 2020). Some agronomically significant genes have been stably integrated and expressed in plastids, and vegetable crops can be used for the production of industrially important products and increase in nutritional value (Yarra, 2020). In vegetable plants such as tomato, eggplant, potato, carrot, cabbage, lettuce, cauliflower, soybean, and bitter melon, chloroplast transformation is successful (Ruhlman et al., 2006; Zhou et al., 2008; Jo et al., 2011; Ding et al., 2016; Yarra, 2020; Chayut et al., 2021), but only transplastomic tomato and lettuce exhibited an accumulation of foreign proteins (Liu et al., 2020; Ling et al., 2021). Plastid genome engineering has potential applications in molecular agriculture to accumulate carotenoids for better nutrition (Bock, 2022; Singhal et al., 2022). For instance, plastid expression of a bacterial LCYB gene triggers conversion of lycopene to β-carotene, thus resulting in quadrupled pro-vitamin A content in tomato fruits (Wurbs et al., 2007).

## Plastid proteome

### Plastid proteomics

The plastome encodes less than 100 ‘autonomous’ genes (Bharadwaj et al., 2019) and remaining 3500 ~ 4000 plastid proteins building and maintaining the functional apparatus are encoded by the nuclear genome (Beck, 2005). The sequential bioprocesses including transcription in the nucleus, translation in the cytosol, the transportation of proteins into the plastid, and protein degradation in the plastid ensure the assembly of the plastid proteome (Egea et al., 2010). High-throughput proteomics is performed upon the tremendous genomics and bioinformatics, and has matured to identify proteins involved in the carotenoids accumulation and the plastid differentiation (Mergner and Kuster, 2022). In addition, various proteomics approaches have been developed and adopted in either whole chloroplast or sub-plastidial fractions (i.e. purified envelope membranes, stroma, and thylakoids) (Subba et al., 2019). There are three main steps in most proteomics technologies, including protein extraction, separation, and identification or quantification (Mergner and Kuster, 2022). These tools paved the way for high-throughput protein localization, quantification, and post-translational modifications (PTMs) analysis in plastids. For example, the Plant Protein Database (http://ppdb.tc.cornell.edu/default.aspx, Sun et al., 2009) presents the most exhaustive plastid proteome available online. And AT_CHLORO is a comprehensive chloroplast proteome database with sub-plastidial localization and curated information on envelope proteins (Bruley et al., 2012). Plastid proteome reveals the general and basic metabolism and function of the chromoplasts and meanwhile bring new insights into its uniqueness.

**Figure 2:**
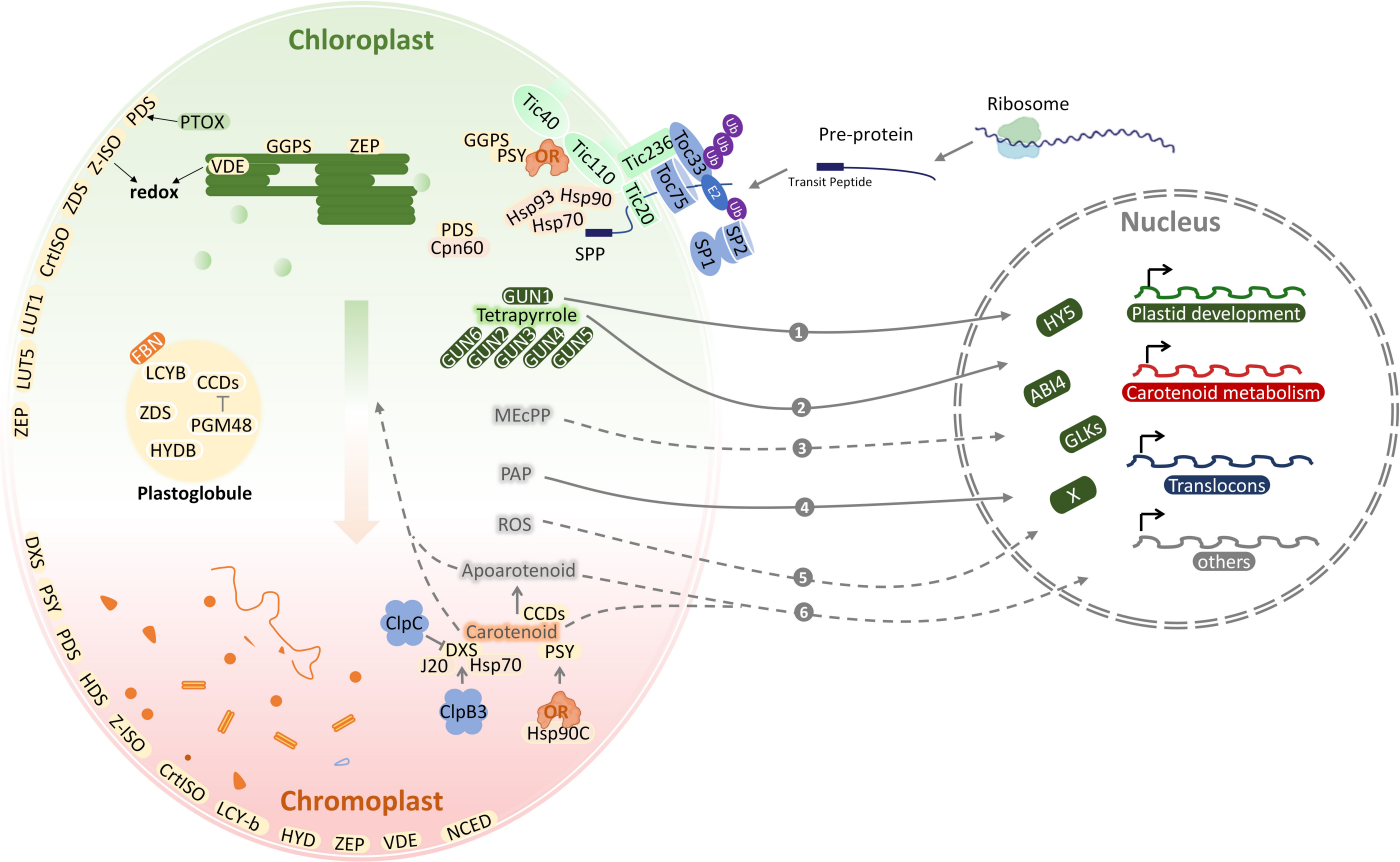
The coordination of plastid development and carotenoid metabolism. Carotenoid metabolic sink is enhanced during chloroplast-to-chromoplast transition. Locations of carotenoid pathway enzymes within chloroplasts and chromoplasts are shown based on subplastidial proteomic identification. Nucleus-encoded chloroplast proteins are synthesized in the cytosol as pre-proteins and post-translationally imported into the chloroplast through the TOC/TIC translocons. With the integration of retrograde communication with nucleus and protein quality control, the plastid development and carotenoid metabolism adapt to each other. The various molecules involved in retrograde signaling are numbered. ABI4, abscisic acid-insensitive 4; BCH, β-carotene hydrolase; CCD, carotenoid cleavage dioxygenase; Clp, Casein lytic proteinase; Cpn60, chaperonin 60; CrtISO, carotene isomerase; DXS, deoxy-D-xylulose 5-phosphate synthase; E2, E2 conjugase; FBN, Fibrillins; GLK, Golden2-like; GUN, genomes uncoupled; Hsp, Heat shock proteins; HY5, long hypocytol 5; HYDB, β-carotene hydroxylase; IEM, inner envelope membrane; J20, J-Protein 20; MEcPP, methylerythritol cyclodiphosphate; NCED, 9-cis-epoxycarotenoid dioxygenase; OR, ORANGE protein; PAP, 3-phosphoadenosine 5 -phosphate; PGM48, plastoglobule-localized metallopeptidase; PTOX, plastid terminal oxidase; ROS, reactive oxygen species; SP, suppressor of ppi1; SPP, stromal processing peptidase; Tic, translocon at the inner envelope membrane of chloroplasts; Toc, translocon at the outer envelope membrane of chloroplasts; Ub, ubiquitin; VDE, violaxanthin de-epoxidase; ZEP, zeaxanthin epoxidase.

The study of the proteomes in heterotrophic plastids is limited and mainly performed in wheat amyloplasts (Ma et al., 2018), rice etioplasts (von Zychlinski et al., 2005), citrus elaioplast (Zhu et al., 2018) and tobacco proplastids (Sakai et al., 1992). Proteomic analysis of chloroplasts has been extensively studied for many years and has illustrated carotenoid function in chloroplasts (**Figure 2**) (Joyard et al., 2009). As the major plastids that synthesize and store carotenoids, chromoplasts contain a wide variety of carotenoids in different species and organs (Sun et al., 2018). A number of proteomic studies have been conducted on chromoplasts of fruits and vegetables, which identified 1170 proteins from watermelon (*Citrullus lanatus*), 1581 proteins from red papaya (*Carica papaya*), 493 proteins from sweet orange (*Citrus sinensis*), 1891 proteins from carrot (*Daucus carota*), 2262 proteins from orange curd cauliflower (*Brassica oleracea*), 1752 proteins from red bell pepper (*Capsicum annuum*), and 988 proteins from tomato (*Solanum lycopersicum*) (Barsan et al., 2010; Wang et al., 2013). Although chromoplast from different horticulture species share similar carotenoids metabolic processes, the distinctive protein distribution patterns were observed in chromoplast proteome, which varies in the morphology of carotenoid-accumulating substructures (Sadali et al., 2019). In addition, 1937 proteins were identified during tomato chloroplast-chromoplast transition (Barsan et al., 2012). Chromoplast proteins differ considerably in relative abundance from the chloroplast proteome, suggesting metabolic processes specific to chromoplasts. As chromoplasts from diverse fruits are derived either from chloroplasts or other non-coloured plastids and possess specific carotenoid-accumulating substructures, chromoplast proteomes from different crops should possess unique characteristics (Ytterberg et al., 2006). Plastoglobule protein profiling from pepper fruit chromoplasts and from *Arabidopsis* leaf chloroplast has been performed (Nacir and Brehelin, 2013; Wojtowicz and Gieczewska, 2020), yielding around 20 proteins (Rottet et al., 2015), including PSY, ZDS, LCYB, BCH1, BCH2, which suggest plastoglobules are not only involved in the carotenoid biosynthesis but also the carotenoids sequestration (Diretto et al., 2020). Fibrillins (FBNs), which are structural proteins for plastoglobules formation, are identified in various plastids that produce plastoglobules, including chromoplasts, chloroplasts, and elaioplasts (Schwenkert et al., 2018; Zhu et al., 2018). There is a fibril structure in plastoglobules of fruits and petals that accumulate carotenoids and enzymes for carotenoid metabolism (Kim and Kim, 2022).

### Plastid protein localization and transport

The structural differentiation of the plastids is characterized by the sharp and continuous decrease of thylakoid proteins, while the envelope and matrix proteins remain stable (Majeran et al., 2012). This is related to the photosynthetic mechanism of thylakoids, the destruction of photosystem biogenesis, and the loss of the plastid division mechanism (Galpaz et al., 2008; Joyard et al., 2009). Most of the work related to the plastid proteome has been conducted on chloroplasts, including sub-organelle protein localization for the thylakoid and lumen as well as the stroma, envelope, and plastoglobules (Wojtowicz and Gieczewska, 2020; Zhu et al., 2022). Sub-plastidial proteomic studies locate most carotenogenic enzymes in chloroplast envelopes (**Figure 2**) (Vishnevetsky et al., 1999; Michel et al., 2021), with ZEP being found both in envelopes and thylakoids, whereas VDE appears only in the latter (Sun et al., 2018). According to proteomic analysis of chloroplasts, lycopene cyclization, responsible for the formation of β-carotene, lutein, and zeaxanthin, occurs in the envelope membranes (Facchinelli and Weber, 2011). As a whole, these data demonstrate that chloroplast envelope membranes participate in carotenoid biosynthesis and metabolism, including the synthesis of precursors from signaling molecules such as ABA (Dong et al., 2015; Wang et al., 2021).

Many proteins translocate from their place of synthesis to plastids or shuttle between different cellular compartments as part of a signal (Thomson et al., 2020). Posttranslational modifications, protein interactions, changes in conformation can modulate localization signals under different conditions (Mergner and Kuster, 2022). In addition, protein targeting mechanisms have been advanced (Zhu et al., 2022). Typically, chloroplast-localized proteins are synthesized as precursors with an N-terminal sign al known as a transit peptide (TP) and delivered to the chloroplast surface by cytosolic chaperones (Fish et al., 2022). Most of these proteins are imported through translocons of the inner and outer membrane of the chloroplast, termed TIC andTOC, respectively (Hoermann et al., 2007). Translocon complexes in envelope membranes facilitate TP entry into the chloroplast *via* translocon recognition (**Figure 2**) (Richardson and Schnell, 2020). A stromal processing peptidase (SPP) then cleaves off the TPs of translocated precursor proteins (pre-proteins) (Kmiec et al., 2014), before final folding and assembly in the stroma or further transport to the thylakoids (Chen et al., 2022). For plants such as Arabidopsis, pea, and tomato, the TOC machinery exists in multiple bv forms and plays a key role in precursor protein recognition (**Figure 2**) (Ganesan and Theg, 2019). This may help to accommodate different proteomes in diverse plastid types, as the majority of proteins in plastids are imported from the cytosol (Fish et al., 2022). It was discovered that this RING-type E3 ligase suppresses the *ppi1* locus 1 (SP1) (Ling et al., 2019), which is a *TOC33* knock-out mutant with pale yellow phenotypes in Arabidopsis thaliana (Thomson et al., 2020). An important mechanism underlying the transformation of plastids from etioplast to chloroplast and chloroplast to gerontoplast transitions involves direct action of the ubiquitin–proteasome system, and is mediated by SP1 (Ling et al., 2019), which is located in the plastid outer envelope membrane. It has recently been shown that the tomato homologs *SP1* and *SP1-like2* (*SPL2*) also play key roles in chloroplast-to-chromoplast transition (**Figure 2**) (Ling et al., 2021). As tomatoes ripen, the chloroplasts of mature green fruit degrade their chlorophyll and gradually convert to chromoplasts that accumulate large amounts of carotenoids including lycopene and β-carotene, the main precursor of vitamin A (Giuliano, 2017; Ling et al., 2021). The alteration of *SlSP1* expression can be attributed to the regulation of plastid protein import by CHLORAD, which in turn regulates the plastid proteome. In agreement with this, knockdown of *SP1* or *SPL2* delayed the process of chromoplast differentiation (Ling et al., 2021). Different components and isoforms of the TIC machinery mediate the translocation of preproteins through the inner envelope membrane in chloroplast. It remains unclear whether they play a role in the discriminative import of specific precursor proteins into chromoplasts or any other type of plastid.

Additionally, a Ycf2-FTSHi ATPase complex, acting as a motor for pulling preproteins across the inner envelope membrane, was identified as being associated with the TIC machinery (Xing et al., 2022). Orf2971, the ortholog of Ycf2 and, is encoded by the largest gene in chloroplast genome of *Chlamydomonas reinhardtii* (*Chlamydomonas* throughout) and directly associated with the protein import machinery to ensure the quality of proteins targeted to the chloroplast (Xing et al., 2022). Particularly, Orf2971 interacts with CrTic214 (also called Orf1995 or Ycf1), a protein involved in chloroplast protein translocation, as well as with chaperones like Hsp70 and Cpn60, which direct protein folding or assembly (Bonk et al., 1996; Bonk et al., 1997). TOC33 belong to the TOC complex, which contains cytosol-projecting GTPase domains that bind to preprotein TPs (Kessler and Schnell, 2006). TOC75, the β-barrel protein, is responsible for translocating pre-proteins through the outer envelope membrane. A continuous chaperoned passage for preprotein translocation across the intermembrane space is provided by TIC236 and POTRA domains of TOC75 (Chen Y. L. et al., 2018). Other mechanisms may ensure inward transport, such as increased affinity for transit-peptides or stromal ATPase motors (Ling et al., 2019). Different components and isoforms of the TIC machinery mediate the translocation of preproteins through the inner envelope membrane. It remains unclear whether they play a role in the specific import of certain precursor proteins into chromoplasts or other kinds of plastid.

### Key proteins and regulatory mechanism identified from proteomics

Plastid proteome changes are associated with or caused by chromoplast differentiation (Kahlau and Bock, 2008). Proteomic studies have shown that proteins involved in photosynthesis are typically reduced following the chloroplast-to-chromoplast transition, whereas many non-photosynthetic plastid proteins, including those linked to the biosynthesis of carotenoids, vitamins, hormones, and aroma volatiles, are accumulated (Moreno, 2018). Proteomics has identified several key proteins as well as regulatory mechanisms, including protein import, quality control, and structural proteins. After protein introduction, nuclear encoded plastid protein transport peptides are cleaved by specific proteases, and mature proteins are folded, assembled into complexes, or transmitted to their target sites in plastids through the complex network of plastid chaperones (**Figure 2**) (Torres-Montilla and Rodriguez-Concepcion, 2021). Molecular chaperones and proteases also form a protein quality control (PQC) system to ensure the stability, refolding or degradation of mature proteins (Rochaix, 2022). Plastid PQC components include chaperones, such as heat shock proteins Cpn60, Hsp70, and Hsp100, and proteases Clp, Lon, Deg, and FstH (Torres-Montilla and Rodriguez-Concepcion, 2021). Our understanding of the PQC system mainly comes from the model of chloroplast. Genetic manipulation of these components in tomato and other crops changes their carotenoid content and affects chromoplast differentiation (Torres-Montilla and Rodriguez-Concepcion, 2021).

Among the molecular chaperones, a well-studied one affecting chromoplasts differentiation is ORANGE (Lopez et al., 2008), and increased carotenoid accumulation was observed in *OR* mutants of cauliflower and melon (Lu et al., 2006; Chayut et al., 2021). Heterologous overexpression of *OR* increases carotenoid accumulation by promoting chromoplasts differentiation (Lopez et al., 2008; Welsch et al., 2019). PSY is the first rate-limiting enzyme in carotenoid metabolism, and OR acts as a molecular chaperone to prevent PSY degradation (Lu et al., 2006). In addition, OR also affects carotenoid accumulation and chromoplasts differentiation through other mechanisms (Giuliano, 2017). It plays a role in protein input by physically interacting with many components in the TIC machine, including TIC40 and TIC110 (Yuan et al., 2021). OR also interferes with the interaction between TIC40 and TIC110 by reducing the turnover of TIC40 (Fish et al., 2022), and reducing the binding of pre-protein and TIC110, thereby carrying out protein translocation and processing (Yuan et al., 2021). The OR of a “golden SNP” (OR^His^) in melon fruit prevents the conversion of β-carotene to downstream products (Chayut et al., 2017; Giuliano, 2017). OR^His^ promotes chromoplast differentiation when overexpressed in tomato fruit (Yazdani et al., 2019). Increased chromoplast number and size enhances carotenoid metabolic strength, such as increased total carotenoid levels in fruits of tomato *high pigment1* (*hp1*) mutant (Galpaz et al., 2008; Kilambi et al., 2013). In addition to promoting carotenoid biosynthesis and chromoplasts biogenesis, OR^His^ also functions in regulating the number of chromoplast (Sun et al., 2020). In *Arabidopsis*, both ARC3 and PARC6 are key regulators of plastid division, and OR^His^ limits the number of chromoplasts by interacting with ARC3 to interfere with its binding to PARC6 (Sun et al., 2020). Overexpression of OR^His^ did not alter the number or ultrastructure of chloroplasts, and the direct interaction of OR with TCP14 inhibited its transactivation activity and ultimately suppressed the etioplast-to-chloroplast transition during desulfurization in *Arabidopsis* seedlings (Sun et al., 2020). The study of molecular chaperone OR has paved the way for further unravelling the molecular mechanism of carotenoid accumulation in plastids. In plastids, the Clp protease system is the main mechanism for removing improperly folded proteins and inhibiting toxic protein aggregate formation (Welsch et al., 2018). The Clp complex has two domains: a proteolytic core consisting of two loops formed by the ClpP and ClpR subunits, and a chaperone loop formed by the ClpC and ClpD subunits responsible for transporting substrates into the proteolytic compartment (Welsch et al., 2018). The Clp protease complex controls DXS, PSY and other enzymes involved in carotenoid biosynthetic pathway (Sun et al., 2018). In tobacco, higher levels of carotenoid biosynthesis enzymes were observed in plants with deletions of subunits of the catalytic core of Clp (such as ClpR1) or the chaperone loop (such as ClpC1), suggesting that these enzymes are direct targets of proteases (Welsch et al., 2018). However, carotenoid levels were not increased in leaves of these lines, as defective Clp protease function also affects chloroplast development and thus alters carotenoid deposition sites (D’Andrea et al., 2018). Knockdown of *ClpR1* in tomato fruit inhibited the activity of Clp protease and increased the levels of DXS and PSY proteins in chromoplasts (D’Andrea et al., 2018). *ClpR1*-deficient tomato fruits show increased expression of plastid chaperones genes, with a similar phenomenon observed in *Clp protease-deficient Arabidopsis* mutants, which may be a compensatory mechanism to alleviate protein folding stress due to turnover defects (D’Andrea et al., 2018). *ClpR1*-deficient fruits expressing *OR* displayed a phenotype of β-carotene-rich chromoplast development similar to *OR* overexpression lines (D’Andrea et al., 2018). Chaperone proteins such as OR and proteases such as Clp are closely linked and cooperate with each other to ensure the normal function and differentiation of chromoplasts.

## Plastid metabolome

Plastids are essential biosynthetic organelles. To understand the regulation of the biological processes in plastid, the *in vivo* reactions of metabolites have to be measured separately. The in-depth metabolite profiling has been a pivotal tool to elucidate the environmentally and developmentally dependent dynamics of metabolism. Most metabolic profilings provide an average across different cell types and all sub-cellular compartments. However, the organelles in the different cell types execute distinct metabolic programs and produce unique metabolites. Thus, considering metabolic heterogeneity, it is vital to perform metabolome analysis in organelle resolution. Non-aqueous fractionation (NAF) method has been developed to measure the sub-cellular distribution of metabolites in plant cells by density gradient and has been broadly applied (Geigenberger et al., 2011; Dietz, 2017). For example, 70 metabolites were obtained from sub cellular compartment of Arabidopsis leaves by NAF, including plastids, cytoplasm, vacuoles, mitochondria and peroxisomes (Arrivault et al., 2014) and the cold-induced sugar and amino acid dynamics in chloroplasts of leaf were explored (Nägele and Heyer, 2013; Fürtauer et al., 2016). The metabolites in cytosol, plastids, and vacuole were profiled during apple fruit development with NAF method, demonstrating the localization of sugars and organic acids in vacuole and amino acids in cytosol and the vacuole (Beshir et al., 2019). The lipoprotein particles, plastoglobuli (PGs), are present in different plastids, including chloroplast and chromoplast (**Figure 2**). PGs in chloroplasts function in metabolism of prenyl lipids and jasmonate. Whereas, in chromoplasts, PGs are found to be highly enriched in carotenoid esters by systerm analysis (van Wijk and Kessler, 2017). Still, the attention on plastid metabolites profiling is limited, even though sigle cell sequencing has been rising. Plastid or other organelles in sigle cell level is challenging but essential to dissect the heterogeneity in cellular populations. The transport systems in plastid envelope membrane determines the type and rate of metabolic pathways. With the advanced mutiple omics technologies, the corresponding proteins and the associated metabolites are expected to be elucidated, which will facilitate our understanding of the metabolic networks in plastid and develop strategies for carotenoid biofortification in crops.

## The coordination of plastid signaling and carotenoid metabolism

### Plastid retrograde signaling

The current regulatory mechanisms of carotenoid metabolism include transcription of carotenoid biosynthetic and catabolic genes, translation and activity of the corresponding enzymes, and plastids development for storage. All three aspects are fine coordinated by both developmental signals and environmental stimuli. For example, the transition from etioplasts to chloroplasts during photomorphogenesis is concomitant with carotenoid accumulation to protect the photosynthetic apparatus against photodamage caused by light stress (Gommers and Monte, 2018). Chromoplast with specialized carotenoid-sequestering structures is the most efficient plastid in carotenoid production and storage. The characteristic orange and red colors in ripe tomato and many other fleshy fruits result from the chloroplast-to-chromoplast transition concomitant with chlorophyll degradation and the surge of carotenoids, mainly lycopene and β-carotene. At the transcriptomic level, nuclear genes encoding carotenoid biosynthetic enzymes are up-regulated, whereas those encoding photosynthesis related proteins are down-regulated (Sadali et al., 2019). Still, the fact that what came first: the plastid type is transformed for carotenoids or the carotenoids trigger the plastid transition, is a conundrum. In either case, the effective and timely communication between plastid and nucleus is essential and is governed by different signaling (Moreno et al., 2021). The coordinated action of the nucleus and plastid is ensured by two types of signals: anterograde signals from the nucleus to the plastids, and retrograde signals from plastids to nucleus (Jiang and Dehesh, 2021). The key components of anterograde signaling pathways in carotenoid metabolism in particular is better established than the retrograde signals. This section mainly focuses on the coordination of plastid retrograde signaling and carotenoid metabolism.

Chloroplasts, the hub of all carbon sources, is also a sensor in perceiving developmental signals and environmental stresses. Chloroplast signals harmonize gene expression at transcription and posttranscriptional levels in the nucleo-cytoplasmic compartment. The signaling molecules in response to real-time disruptions and metabolic status in plastid will be transported out to be sensed and transducted in cytosol and nucleus. However, the mechanism by which plastid connect with the nucleus through the cytosol is still not clear. Insights into the plastid-to-nucleus signaling have come from the application of two photobleaching agents, norflurazon (NF, an inhibitor of the carotenoid biosynthesis enzyme phytoene desaturase) and lincomycin (Linc, plastid translation inhibitor). Both NF and Linc treantments repress expression of photosynthesis-associated nuclear genes (PhANGs), such as light-harvesting Chla/β-binding protein (LHCB) (Cottage et al., 2008; Zhao et al., 2018). A set of genomes uncoupled (*gun*) mutants was genetic screened through the rescue of *LHCB* expression after NF treatment (Susek et al., 1993; Mochizuki et al., 2001; Larkin et al., 2003; Koussevitzky et al., 2007; Woodson et al., 2011). Mutant lines *gun 1* to *6* affected tetrapyrrole metabolism. Particularly, *GUN2* to *GUN6* encode enzymes in the tetrapyrrole biosynthesis or metabolism and *GUN1* encodes a chloroplast protein containing a pentatricopeptide repeat (PPR) protein with a small MutS-related domain, which can bind tetrapyrroles and regulate the flow through the tetrapyrrole biosynthesis pathway (Woodson et al., 2011; Zhao et al., 2018; Shimizu et al., 2019). The adapted nuclear gene expression requires transcriptional factors (TF) downstream of plastid signals. Abscisic acid insensitive 4 (ABI4), the “master switch” of ABA, is a nuclear APETALA 2 (AP-2)-type TF and controls the expression of a large number of genes (Finkelstein et al., 1998). Elongated hypocotyl 5 (HY5), one of the potent TFs involved in light response, is a bZIP transcription factor that promotes de-etiolation and chloroplast development. Golden2-like (GLK) synchronize the expression of a suite of chloroplast genes and *PhANGs* including chlorophyll biosynthesis, light harvesting, and carbon fixation genes, contributing to retrograde signal response (Waters et al., 2009). Chloroplast outer membrane-bound plant TF named phd transcription factor with transmembrane domains 1 (PTM1) mediates chloroplast signals, regulating the expression of *PhANGs*. Besides, an increasing number of signaling molecules have been identified to be involved in plastid retrograde signaling transduction, including (1) tetrapyrrole biosynthetic pathway, such as MgProto and heme (Woodson et al., 2011), (2) reactive oxygen species (ROS) in the plastids (Apel and Hirt, 2004), (3) methylerythritol cyclodiphosphate (MEcPP) (Xiao et al., 2012), (4) SAL1-3-phosphoadenosine 5-phosphate (PAP) (Zhao et al., 2019), and (5) apocarotenoids (**Figure 2**).

Carotenoid compositions and concentrations are adjusted in accordance with plant developmental stages and environmental changes. In chloroplast, carotenoids are integral to the light-harvesting apparatus and serve as structural components of photosystems. In contrast to chloroplast, less is studied about the retrograde signals during the differentiation of chromoplast. Massive reprogramming of the nuclear transcriptome during chloroplast-to-chromoplast transition in tomato have been revealed. The transition is achieved by the coordinated assembly of nucleus- and plastid-encoded proteins, as well as the simultaneous incorporation of pigments (chlorophylls and carotenoids). Thus, compared to the ‘green’ signaling molecules, there is a possibility that carotenoids and/or their degradation products also serve as plastidial retrograde communication metabolite. Carotenoid degradation by *CCDs* produces apocarotenoids. The *ζ-carotene desaturase* (*zds/clb5*) exhibits stunted growth and albino seedling phenotypes due to an apocarotenoid signal that alters *PhANG* expression (Avendaño-Vázquez et al., 2014). The plastidial development perturbation in the carotenoid biosynthesis mutant, *carotenoid chloroplast regulation 2* (*ccr2*) indicating the existence of a unique apocarotenoid retrograde signal that functions through PIF3, HY5 and photomorphogenesis repressor deetiolated1 (DET1) (Cazzonelli et al., 2020). β-Cyclocitral, an apocarotenoid produced by oxidative cleavage in chloroplasts, was identified as a protective retrograde metabolite in response to excess illumination *via* the TGAII/Scarecrow-like protein 14 (SCL14) transcription factor (D’Alessandro et al., 2018). The molecular action of β-cyclocitral was initially reported to induce salicylic acid (SA) and subsequent transcriptional activation of gluthathione-S-transferase 5 (GST5) and GST13 (Lv et al., 2015). The other mode of β-cyclocitral action is *via* the induction of PAP accumulation and trigger PAP-mediated retrograde signaling in response to disturbed photosynthetic process. The transcriptome analysis of β-cyclocitral-treated plants revealed a reprogramming in the expression of genes associated with higher photosystem II photochemical efficiency and lower lipid peroxidation (Ramel et al., 2012). In addition, overexpression of a bacterial phytoene synthase gene (*crtB*) caused reduced chloroplast photosynthetic competence, excessive metabolic flux into carotenoid biosynthesis, and induced the transition from chloroplasts to chromoplasts in various tissues of different plant species (Llorente et al., 2020) (**Figure 2**). This demonstrates a retrograde signal emitted from carotenoid-accumulating plastids to facilitate chromoplast formation.

### The effect of the disturbed carotenoid metabolic pathway on plastid development and function

Modification of structural genes and regulators can affect their expression and cause the accumulation of specific carotenoids in plants (Kilambi et al., 2013). For example, the altered content of carotenoid compositions making the yellow, tangerine, orange, and orange-red tomato fruits (Galpaz et al., 2008). The changes of the predominant carotenoids in the disturbed lines are shown in **Table 2**. The general pattern is that silencing of metabolic genes leads to increased accumulation of upstream substrates and decreased levels of downstream products by feedback and feedforward regulation. In tomato, phytoene and lycopene were decreased in *GGPPS* knock-out mutants (Barja et al., 2021). The levels of the predominant carotenoids were elevated in *PSY1* overexpressed tomato fruits and significantly decreased in *PSY1* RNA-i mutant (Aluru et al., 2006; Fraser et al., 2007). PDS-silenced fruits displayed reduced levels of total carotenoids, with phytoene and phytofluene being the most abundant compounds, whereas ZDS-silenced fruits, contained abundant ζ-carotene and traces of neurosporene. However, not all downstream carotenoid composition levels will be reduced by the enzyme gene shut down; some will result in increased lutein and β-carotene contents. *ZISO* silencing resulted in a significant decrease in lycopene and a compensatory increase of phytoene, phytofluene, and ζ-carotene, while downstream compounds, such as lutein, were increased. We are aware of the consequences of carotenoid metabolism disorders and understand that plastids are linked to carotenoid metabolism (Sun et al., 2018; Sun et al., 2022).

**Table 2:** Effects of different carotenoid biosynthetic genes on carotenoid composition and content as well as plastid morphology.

Common name	Organ/tissue	Gene	Approach	Effects on carotenoids	Effects on plastids	Reference
Tomato	Leaf	*GGPPS*	CRISPR-cas9	Decrease: β-carotene	similar number and size of chloroplasts in guard cell; smaller chloroplasts in mesophyll cells	(Barja et al., 2021)
Tomato	Fruit	*GGPPS*	CRISPR-cas9	Decrease: phytoene, lycopene and total carotenoids	NA	
Tomato	Fruit	*PSY1*	Over-expression	Increase: phytoene, lycopene, β-carotene, lutein and total carotenoids	absence of distinct thylakoid membranes in mature green fruit	(Fraser et al., 2007)
Tomato	Fruit	*PSY1*	Mutation	Decrease: phytoene, lycopene, β-carotene and lutein	NA	(Gady et al., 2012)
Oncidium hybrid orchid	Leaf	*PSY*	RNA-i	Decrease: lycopene and lutein	decreased number of plastids in leaves; containing barely any grana, no starch grains and oil droplets, and little thylakoid lamellar structures	(Liu et al., 2014)
Tomato	Fruit	*PDS*	Over-expression	Decrease: phytoene Increase: ζ-carotene, lycopene, β-carotene	NA	(McQuinn et al., 2018)
Arabidopsis	Leaf	*PDS3*	Mutation	Decrease: lutein and β-carotene Increase: phytoene	no mature plastids and no thylakoid membrane system; lost ability to develop into mature chloroplasts	(Qin et al., 2007)
Tomato	Fruit	*ZISO*	VIGS	Decrease: ζ-carotene, lycopene and β-carotene Increase: phytoene and lutein	NA	(Fantini et al., 2013)
Watermelon	Fruit	*ZISO*	Mutation	Decrease: lycopene and β-carotene	NA	(Zhang J. et al., 2022)
Arabidopsis	Leaf	*ZISO*	Mutation	Increase: phytoene	Reduced number of carotenoid globules	(Cazzonelli et al., 2020)
Tomato	Leaf/fruit	*ZDS*	RNA-i	Decrease: lycopene, β-carotene and total carotenoids Increase: phytoene	disrupted thylakoid membrane structure in the leaf; green-white plastid structures in fruit	(Babu et al., 2020)
Tomato	Fruit	*ZDS*	Over-expression	Decrease: lycopene, β-carotene and lutein Increase: phytoene and ζ-carotene	NA	(McQuinn et al., 2020)
Arabidopsis	Leaf	*CRTISO*	Mutation	Decrease: lutein Increase: violaxanthin	lack of prolamellar bodies in etioplasts; less prothylakoid membranes	(Park et al., 2002)
Tomato	Fruit	*CRTISO*	Mutation	Decrease: lycopene, β-carotene and total carotenoids Increase: phytoene and ζ-carotene	NA	(Isaacson et al., 2002)
Sweet potato	Storage roots	*LCYB2*	Over-expression	Increase: β-carotene, lutein and total carotenoid	large numbers of carotenoid globules	(Chen et al., 2018a)

Since plastids are organelles for carotenoid biosynthesis and storage, the levels of diverse carotenoid compositions can also in turn alter plastids development (Sun et al., 2018). When carotenoid metabolic pathways are disrupted, not only the development of carotenoid storage structures in the plastid is affected, but also the function of the plastid as well as the developmental processes are affected (**Table 2**). Modification of key enzyme genes for carotenoid metabolism causes changes in plastid microstructure. It was found that overexpression of the *LCYB2* in potato resulted in an increase in lutein and β-carotene content, as well as an increase in the number of plastid globules (Chen S. et al., 2018; Diretto et al., 2020). Second, the function of plastids is also affected by changes in key enzyme genes. The thylakoid membrane structure was found to be disrupted in the leaf tissues of *ZDS* RNAi plants exposed to sunlight (Babu et al., 2020). The *PSY* RNAi-transgenic orchids exhibit a disappearance of the thylakoid lamellar structure (Liu et al., 2014). Mutation of key enzyme genes for carotenoid metabolism also caused stagnation of plastid development and loss of functional structures. One of the more intensively studied is that mutation of *CRTISO* leads to the disappearance of prolamellar body in the etioplast and the plant cannot synthesize chlorophyll normally for photosynthesis (Park et al., 2002). There is no doubt that carotenoid metabolites influence the substructure, function, and developmental processes of plastids. The mechanism by which metabolites themselves influence plastids remains largely unknown.

## Prospect

Integrating information from multiple omics, such as genomics, transcriptomics, epigenomics, proteomics, metabolics, phenomics, next-generation sequencing, and advanced bioinformatics, as a systems biology approach may enable better understanding of metabolic regulatory networks and key components in carotenoid metabolism. Therefore, the plastid “omic” response to the developmental and environmental signals should be further explored. Chromoplast is critically important for carotenoid accumulation in many agricultural plants. In comparison with the understanding of chloroplast retrograde regulatory mechanisms, less is known about its retrograde signaling pathway. Therefore, new strategies for carotenoid biofortification may be developed for human nutrition and health by gain more regulatory components involved in chromoplast biogenesis. Finally, we believe that, the comprehensive utilization of omics methods will help us further reveal the internal regulation of carotenoid accumulation in plastid and benefit biology researchers and breeders in low-cost, scalable, safe, and environmentally friendly molecular farming.

## Author contributions

LL, QW, and YL conceived the idea of the review and prepared the initial outline and the first draft. YL, YJ, YM, and LL gathered the literature and contributed to writing the different sections. FM, ZS, TW, JZ, and QW critically revised the manuscript. All authors contributed to the article and approved the submitted version.

## Funding

This work was supported by National Natural Science Foundation of China (Key Program, No. 31830078; No. 32002024) and Zhejiang Provincial Natural Science Foundation of China (No. LY22C150001).

## Conflict of interest

The authors declare that the research was conducted in the absence of any commercial or financial relationships that could be construed as a potential conflict of interest.

## Publisher’s note

All claims expressed in this article are solely those of the authors and do not necessarily represent those of their affiliated organizations, or those of the publisher, the editors and the reviewers. Any product that may be evaluated in this article, or claim that may be made by its manufacturer, is not guaranteed or endorsed by the publisher.
